# Principal-Oscillation-Pattern Analysis of Gene Expression

**DOI:** 10.1371/journal.pone.0028805

**Published:** 2012-01-10

**Authors:** Daifeng Wang, Ari Arapostathis, Claus O. Wilke, Mia K. Markey

**Affiliations:** 1 Department of Electrical and Computer Engineering, The University of Texas at Austin, Austin, Texas, United States of America; 2 Section of Integrative Biology, Center for Computational Biology and Bioinformatics and Institute for Cellular and Molecular Biology, The University of Texas at Austin, Austin, Texas, United States of America; 3 University of Texas Biomedical Informatics Lab and Department of Biomedical Engineering, The University of Texas at Austin, Austin, Texas, United States of America; Hemocentro de Ribeirão Preto, HC-FMRP-USP, Brazil

## Abstract

Principal-oscillation-pattern (POP) analysis is a multivariate and systematic technique for identifying the dynamic characteristics of a system from time-series data. In this study, we demonstrate the first application of POP analysis to genome-wide time-series gene-expression data. We use POP analysis to infer oscillation patterns in gene expression. Typically, a genomic system matrix cannot be directly estimated because the number of genes is usually much larger than the number of time points in a genomic study. Thus, we first identify the POPs of the eigen-genomic system that consists of the first few significant eigengenes obtained by singular value decomposition. By using the linear relationship between eigengenes and genes, we then infer the POPs of the genes. Both simulation data and real-world data are used in this study to demonstrate the applicability of POP analysis to genomic data. We show that POP analysis not only compares favorably with experiments and existing computational methods, but that it also provides complementary information relative to other approaches.

## Introduction

Genes whose expression varies differentially and periodically over the cell cycle have been identified by both experimental and computational methods [1], [2], [3], [4], [5], [6]. Existing methods analyze individual genes or small-scale gene sets; in contrast, our goal is a systematic, multivariate method for analysis of genome-wide gene-expression data. A graphical approach has been applied to model gene expression data systematically in [7], but it does not identify the genome-wide dynamic patterns such as oscillation patterns. Principal-oscillation-pattern (POP) analysis is a data-driven multivariate and systematic technique for identifying the dynamic characteristics of a system using dynamic system equations. It has been widely used to analyze climate data in the geosciences [8], but to the best of our knowledge, this is the first time that POP analysis has been applied to identify oscillation patterns in gene expression.

Typically, the dynamics of a genomic system are too complicated to be known explicitly. In POP analysis, a complex system is linearized using a set of first order ordinary differential equations (ODEs). These ODEs correspond to the state equation in systems theory; their parameters can be inferred from data. The state equation with perturbations has been applied to model gene expression in [9], but a typical genome-wide gene expression dataset does not reveal the perturbation signals explicitly. Moreover, the method in [9] did not analyze dynamic characteristics from the state equations to identify the genes that express differentially and periodically over the cell cycle. However, POP analysis identifies the dynamic patterns of the genomic system directly from the eigenvalues and eigenvectors of the system matrix.

However, genome-wide gene-expression data sets normally have a limited number of time samples. Since the number of time samples is much fewer than the number of genes, estimation of the genomic system matrix is underdetermined. In order to solve this problem, we use the idea of dimensionality reduction to construct an eigen-genomic system that consists of significant eigengenes calculated from the singular value decomposition (SVD) [10]. We obtain the POPs for the eigen-genomic system, and then make use of the linear relationship between the eigen-genomic system and the genomic system to infer the POPs of the genomic system.

We evaluate the applicability of POP analysis to genomic systems using both simulation and real-world datasets. Using simulation data, we check the capability of POP analysis to recover the oscillation amplitudes and phases defined by the simulation parameters. Using real-world data, we compare POP analysis with both the results of experiments and existing computational methods [1], [2], [3], [4], [5], . We demonstrate that the systematic, multivariate approach of POP analysis can accurately identify genes that are differentially and periodically expressed across the cell cycle.

## Methods

We model gene expression data from a system point of view; i.e., the genome-wide time-series gene-expression data 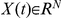 for 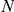 genes at time-points 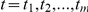 is expressed as a matrix first-order ordinary differential equation, also known as the state equation in systems theory, as follows:



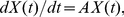
(1)


where 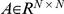 is the genomic system matrix, which models how the current genomic state *X*(*t*) affects the state change rate *dX*(*t*)/*dt*, and also encapsulates the dynamic characteristics of the genomic system, and in this paper, we denote as 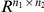 the set of 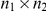 real matrices.

Estimating the genomic system matrix 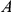 is an underdetermined problem since the number of time samples is typically much less than the number of genes. Instead of estimating the genomic system matrix, the eigen-genomic system matrix, denoted as 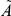, is estimated. The eigen-genomic system is introduced in Eigen-genomic System Dynamic pattern Analysis (ESDA) in [11]. The eigen-genomic system consists of the first few significant eigengenes. The eigengenes can be calculated from the singular value decomposition (SVD) of the gene-expression data matrix. The significant eigengenes are defined to be those that explain most of the covariance of the gene-expression data matrix.

### Eigen-genomic system

In this section, we introduce to calculate the eigen-genomic system matrix. The singular value decomposition (SVD) of 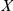
is denoted as svd(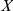)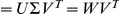. We denote the expression of first 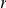 significant eigengenes at time 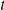 as 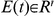, which are the first 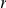 rows of 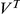. By the linear relationship between gene expressions and eigengene expressions in ESDA, we know that
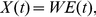
(2)


where 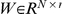 is the coefficient matrix of genes on the first *r* significant eigengenes.

By Equation (1) and Equation (2), the eigen-genomic system matrix 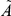 satisfies 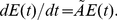
(3)


The relationship between the genomic system matrix 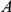 and the eigen-genomic system matrix 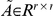 is given by



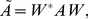
(4)


where 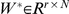 is the pseudo-inverse of 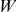.

The eigen-genomic system equation is given by Equation (3). After discretizing it into a difference equation, we obtain



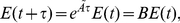
(5)


where *τ* is the time interval between measurement time points and 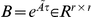.

We can estimate 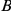 from the eigengene expressions 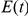 at 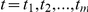 as follows:



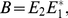
(6)


where 
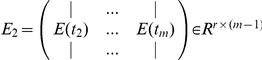
, 
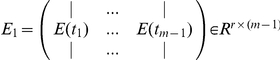
,and 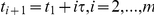.

### POP analysis

By Equation (5), the eigengene expression 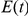 can be decomposed as the linear combination of eigenvectors of 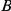 as follows:



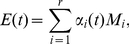
(7)


where 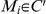 is the 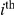 eigenvector of 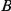, and 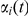 is the coefficient of 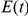 on 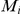.

The coefficient satisfies the dynamic equation:



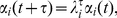
(8)


where 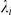 is the eigenvalue of 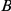. Thus, the coefficient 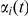 can be calculated as



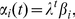
(9)


where 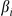 is a scaling factor. Without loss of generality, we assume that 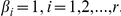


The eigen-genomic system matrix 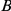 is not necessarily symmetric, so the eigenvalues of 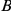 may be complex. Thus, if 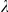 is an eigenvalue of 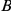 with its eigenvector 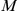, then its conjugate, 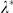 is also an eigenvalue of 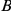 with eigenvector 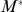.

For a complex conjugate pair of eigenvalues, 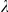 and 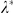, we let 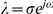, where 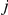 is the imaginary unit. The real part of their eigenvectors is denoted as 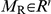, and the imaginary part of their eigenvectors is denoted as 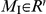.

After summing the terms of the complex conjugate eigenvectors in Equation (7), their sum, denoted as 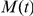, is given by
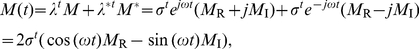
(10)


which shows that the oscillation with frequency *ω* is driven by the patterns 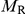 and 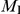. Thus, 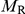 and 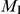 are referred to as the principal oscillation patterns (POPs) of the eigen-genomic system.

By Equation (2), the relationship between gene expression and eigengene expression is linear, so gene expression 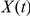 is also a linear summation of 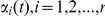. The portion of the summation of the coefficients corresponding to the POPs, 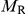 and 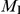 of the eigen-genomic system to 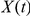, denoted as 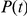, is given by



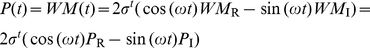
(11)


where 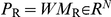 and 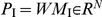 are referred to as the POPs of the genomic system. They drive the oscillation process with the angular frequency *ω*; i.e., the period  = 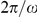.

From a system point of view, the oscillation part of a genomic system is a periodic process starting from 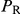 to 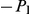 to 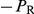 to 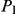, and then back to 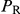 (as shown in Figure S1).

**Figure 1:**
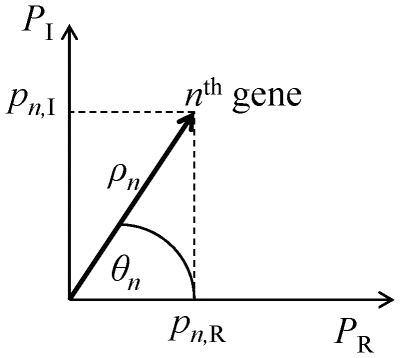
POP amplitude and phase. For the 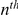 gene, the 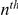 element of a POP represents the coefficient of the 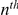 gene on the POP. Its coefficients of the POPs, 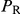 and 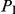 , are denoted as 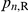 and 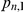, respectively. We convert the coefficient pair (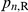,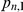) into polar coordinates (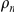,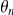), where 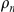 represents the POP amplitude and 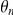 represents the POP phase. A high POP amplitude means that the gene expression level oscillates strongly with the angular frequency *ω*. The POP phase unveils the stage at which gene expression achieves its peak value.

For an individual gene, for example, the 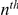 element of a POP represents the coefficient of the 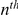 gene on this POP. We denote its coefficients on the POPs, 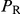 and 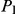, as 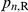 and 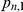, respectively. We convert the coefficient pair (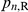,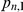) into polar coordinates (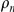,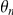) as follows:



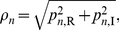
(12)




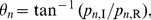
(13)


where 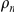 represents the oscillation amplitude of the POPs, and 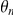 represents the oscillation phase of the POPs. A high POP amplitude 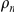 means that the gene expression level oscillates strongly with the angular frequency *ω* as shown in Figure 1. The POP phase 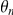 unveils the stage at which the gene expression achieves its peak value.

### Overview of Analysis

We now perform a series of analyses to determine the strengths and limitations of POP analysis as applied to gene expression data:

- Can POP analysis recover known periodic features of a genomic system? To address this question, we apply POP analysis to simulation data. At the system level, we check if the period of the POPs recovers the one defined in the simulated oscillation process. At the gene level, we check if the amplitudes of the POPs are highly positively correlated with the simulated amplitudes of the oscillating strengths, and likewise if the phases of the POPs match the simulated phases.
- What is the sensitivity of POP analysis, i.e., for genes that have been experimentally verified as being periodically expressed, does POP analysis identify them as periodically expressed? To address this question, we examine the results of POP analysis on genes that are experimentally considered to be periodic based on previously reported experimental investigations of gene expression across the cell cycle.
- What is the specificity of POP analysis, i.e., does POP analysis falsely identify genes as periodic that are not actually periodically expressed? To address this question, we examine the results of POP analysis on genes that have never been identified by either previous experiments or existing computational methods as periodic across the cell cycle.
- Can POP analysis identify genes that are likely to be periodically expressed but that were missed in previously reported previous experiments? To address this question, we examine annotations [12] of genes that POP analysis identifies as periodically expressed across the cell cycle but that previous experimental methods don't identify as such.
- Can POP analysis identify genes that are unlikely to be periodically expressed across the cycle, yet were previously reported as such by previous experiments? To address this question, we examine annotations [12] of genes that POP analysis identifies as probably not periodically expressed but that experimental methods identify as periodically expressed across the cell cycle.
- How does POP analysis compare with existing computational methods for identifying periodically expressed genes? To address this question, we evaluate the results of POP analysis relative to existing computational methods [1], [2], [3], [4], [5], [6].

### Simulation data

We simulate the time series expression of each gene using the following first order differential equation, which is widely used in modeling gene-expression data:



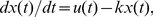
(14)


where 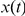 is the gene expression at time 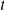, 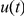 is the transcription rate also known as the production rate, and 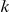 is the decay rate constant. Thus, the gene-expression change rate 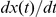 is equal to the difference between the production rate 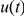 and the degradation rate 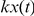. The production rate 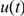 drives the oscillation of 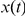. If 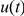 is zero, then 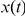 will be a decay process. If 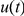 is oscillating, then 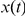 will oscillate as well.

Therefore, using Equation (14), we obtain simulated expressions of 4000 genes at 0,7,14, ... ,119 minutes. These time points were selected to match those of a widely used budding-yeast cell-cycle data set with *α*-factor-based synchronization [5]. We generate the simulated decay half-lives using the lognormal distribution that fit the experimental measurements for mRNA decay half-lives in [13], and obtain the decay rate constant 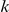 using 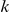  = -ln(2)/half-life. We let the production rate, 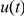, which generates the oscillation process, be 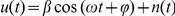. We set the angular frequency, *ω,* as *ω* = 2*π*/30, which corresponds to a 30 minute period of the oscillation process. The simulated phase 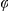 is a random number uniformly distributed on [0, 2*π*]. The simulated amplitude 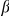 is also a random number uniformly distributed on [0, 0.1] such that simulated expressions are positive. Ten-percent Gaussian noise, 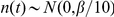, is added to the production rate such that the first five significant eigengenes explain at least 98% covariance of simulated gene expressions.

### Real-world data

We apply POP analysis to a widely studied budding-yeast (*Saccharomyces cerevisiae*) gene-expression dataset with *α*-factor-based synchronization [5]. The state equation (1) assumes that the genomic system matrix is constant, so the estimate of 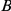 by Equation (6), which describes the dynamic evolution between adjacent time samples of eigen-genomic system, requires that we have an equal time sampling interval. The gene expressions with *α*-factor-based synchronization were measured at *t* = 0, 7, 14, … , 119 minutes covering two cell cycles of around 120 minutes with an equal time sampling interval of 7 minutes [5]. We obtain 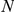 = 4598 genes with no more than three missing samples and ratios of Mean Intensity to Median Background Intensity in both Channel 1 and Channel 2 being greater than 1.5. We estimate the missing samples using the singular value decomposition method as in [10], and normalize time series expression data for each gene such that its norm of expression levels at all time samples is equal to one.

## Results

### Simulation data

We select the first five significant eigengenes to comprise the eigen-genomic system of the simulated data. The eigenvalues of the eigen-genomic system matrix are summarized in Table S1. The first and second eigenvalues are complex conjugate, which correspond to the POPs with a period of 30 minutes that recover the period defined by the simulation parameters. The third, fourth, and fifth eigenvalues are real, so they do not represent the oscillation process but rather the slow, median, and fast decay processes of the system, which are discussed in [11].

**Figure 2:**
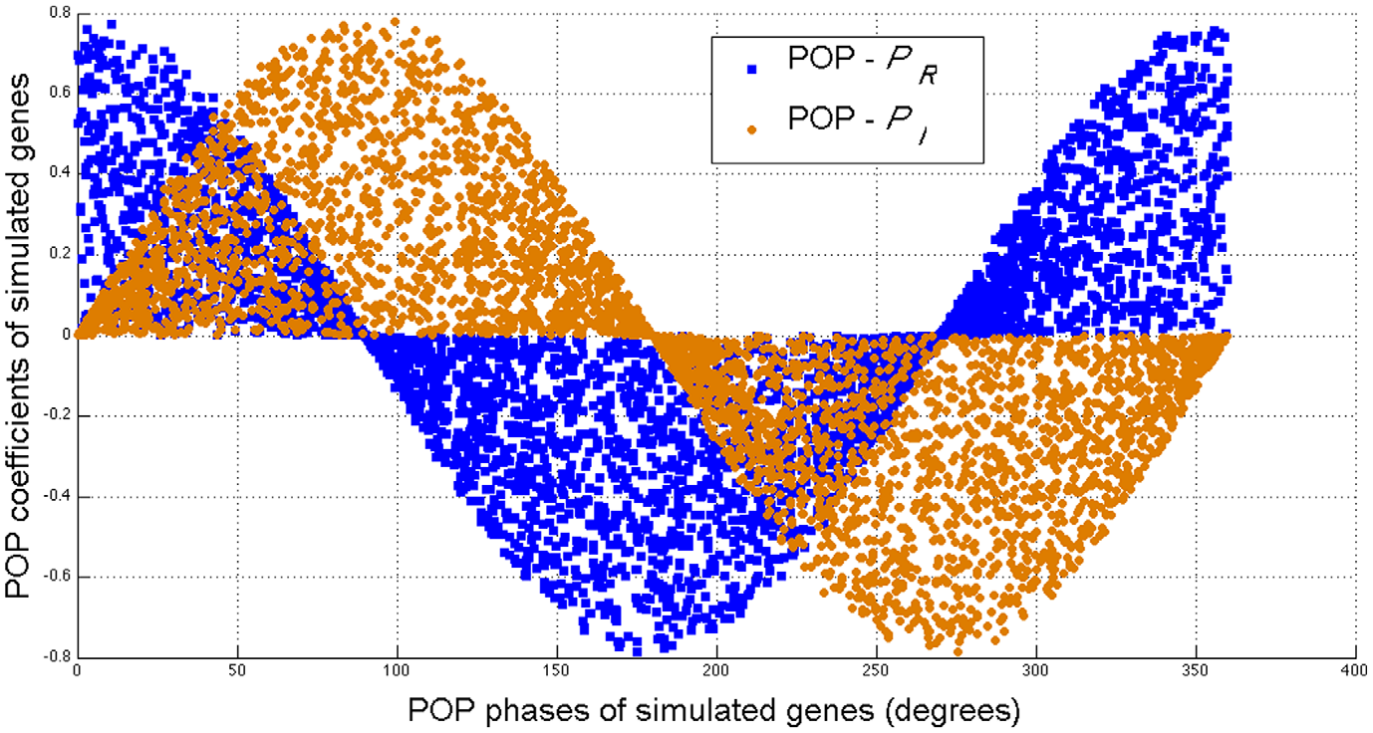
POPs of the simulated genomic system. The horizontal axis represents the POP phases of simulated genes, which unveils when the expression levels of the simulated genes peak. The vertical axis represents the coefficients of simulated genes on each POP. Over 360 degrees that go through a period of 30 minutes, the envelopes of 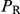 and 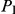 consisting the genes that have large POP coefficients on either POPs, are the cosine and the sine waves, respectively, which shows that 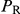 drives the cosine oscillation, and 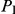 drives the sine oscillation of genomic system.

Thus, we focus on the POPs corresponding to the first and second eigenvalues. These two POPs, 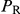
and 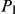, are plotted in Figure 2. The horizontal axis represents the POP phases of the simulated genes in degrees, which unveils when the expression levels of the simulated genes peak. The vertical axis represents the coefficients of simulated genes on each POP. Over 360 degrees, i.e., over a period of 30 minutes, the envelopes of 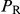 and 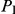 are cosine and the sine waves, respectively, which shows that 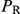 drives the cosine oscillation, and 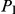 drives the sine oscillation of genomic system. The envelopes of 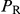 and 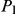 are determined by the genes that have large POP coefficients on these POPs. If a gene has a large coefficient value for either POP at a certain phase, it means that the oscillation pattern of the gene's expression is strong at this phase.

### Pearson correlation between POP amplitudes and simulation amplitudes

We investigate the relationship between the amplitudes defined by the simulation parameters and the amplitudes extracted from the simulated data via POP analysis. The Pearson correlation between the simulated and POP amplitudes over 4000 simulated genes is 0.99 with *p*<0.01 (Figure S2). Thus, the POP amplitudes recover the oscillation amplitudes defined by the simulation parameters. A high POP amplitude of a gene means that its expression level is strongly oscillating with a period of 30 minutes.

### Pearson correlation between POP phase and simulation phases

The phases extracted by POP analysis recover the phases defined by the simulation parameters; the Pearson correlation of the sine values of their phases is 0.96 with *p*<0.01 (Figure S3). So, the POP phase of a simulated gene reflects the phase of the oscillation process in its expression.

### Budding yeast (*Saccharomyces cerevisiae*) cell cycle expression data

**Figure 3:**
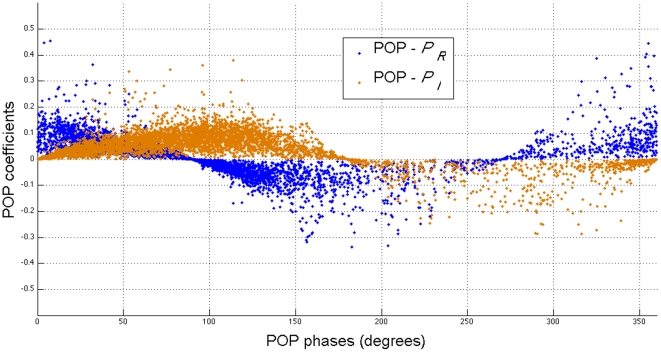
POPs of the real genomic system, budding yeast genomic system. The horizontal axis represents the phases of genes in degree on each POP. The vertical axis represents the coefficients of genes on each POP. The 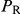 and 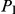 have approximately cosine and sine envelopes, respectively, which is driven by genes around envelopes that have large values of POP coefficients. Thus, these genes have strong oscillation patterns.

We choose the first five significant eigengenes as the eigen-genomic system of the budding yeast since they capture more than 98% of the covariance of the data matrix [10]. The eigen-genomic system matrix of the budding yeast has eigenvalues (as shown in Table S2). The first and second complex conjugate eigenvalues correspond to POPs with a period of 65.7 minutes, which falls into the estimated period of the cell cycle (66±11 minutes) of budding yeast with *α*-factor-based synchronization [5], [14]. We plot POP coefficients versus POP phases for all 4598 genes in Figure 3 (POP amplitudes and phases are included in Table S3). The 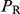 and 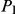 have approximately cosine and sine envelopes, respectively, which are driven by genes that have large values of the POP coefficients. Thus, these genes have strong oscillation patterns.

### Genes periodically expressed across the cell cycle have high POP amplitudes

In order to assess the sensitivity of POP analysis for identifying periodically expressed genes, we examine the results of POP analysis on genes that are experimentally considered to be periodic based on previously reported experimental investigations of gene expression across the cell cycle. In our model, a gene that is periodically expressed across the cell cycle should have a production rate in Equation (14) that has a strong oscillating process with a period matching the length of the cell cycle. Thus, we investigate whether the mean POP amplitude of genes that are known to be periodically expressed across the cell cycle is higher than the mean POP amplitude of other genes.

As summarized in [1], small-scale experiments have measured expression level changes or investigated cell cycle transcription factors for individual genes. Throughout this paper, we refer to methods that infer cell-cycle genes by computational means from genome-wide gene-expression time-series as ‘computational methods’, and we refer to methods that infer cell-cycle genes by identifying periodically expressions in small-scale experiments, or promoters of genes bound by known cell cycle transcription factors, as ‘experimental methods’. Experimental methods have identified a total of 465 cell-cycle genes, including 113 genes that are expressed periodically across cell cycle [2], [5], and 402 genes that are bound by known cell-cycle transcription factors at their promoters [15], [16], which drive the oscillation processes of expression in transcription rate in Equation (14), and, thus, can be inferred to be periodically expressed. We compare the POP amplitudes of these genes to the POP amplitudes of other genes not known to be modulated by the cell cycle. Out of 4598 genes reported in the budding yeast dataset with *α*-factor-based synchronization, there are 344 cell cycle genes identified by experimental methods. The mean of the POP amplitudes of these 344 ‘cell cycle’ genes (0.12) is significantly (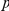
<0.01) greater than the mean of the POP amplitudes of the rest of the genes (0.07) according to a two-sample t-test with unequal variances. (Their variances are not statistically equivalent, F test, 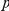
<0.01.) In addition, a permutation test [17] also indicates that the mean of the POP amplitudes of these 344 ‘cell cycle’ genes is statistically significantly (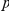
<0.01) greater than the mean of the POP amplitudes of the rest of the genes. The permutation test is implemented as follows: we randomly select 344 genes without replacement, and calculate the difference between the mean of their POP amplitudes and the mean of the POP amplitudes of the rest of the genes. We repeat this random selection 10,000 times, and record the resulting 10,000 differences of the means. The 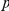-value is calculated as the proportion of the differences of the means that are greater than or equal to the difference of the means observed for actual 344 ‘cell cycle’ genes compared to the rest of the genes.

### Genes maximally expressed in the same phase of the cell cycle have similar POP phases

**Figure 4:**
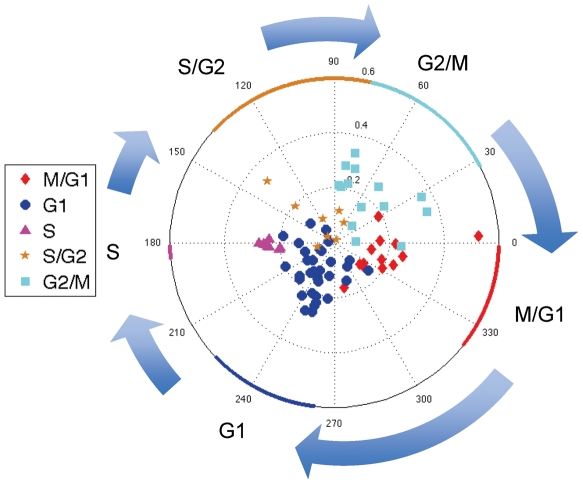
POPs in polar coordinates for experimentally identified ‘cell cycle’ genes. These genes are previously by experimental methods classified into five clusters based on the timing of their maximal expression in different phases of the cell cycle: G1(round), S(triangle), S/G2(star), G2/M(square) and M/G1(diamond). The median POP phases of the five experimentally defined clusters are 331.4° (S/G2), 266.6° (G2/M), 182.4° (M/G1), 102.0° (G1), and 32.4° (S). Genes classified as maximally expressed in the same cell cycle phase have similar POP phases. Different POP phases correspond to different phases of the cell cycle.

In our model, the POP phase should unveil the point in the cell cycle at which a gene reaches its peak expression. Thus, we investigate the correspondences among POP phases of genes that are maximally expressed at the same cell-cycle phases according to reports from previous experiments. In this data set, there are 75 genes that have been classified into five clusters, G1, S, S/G2, G2/M and M/G1, according to experimental investigations of the genes' transcriptional activities [5]. Some genes are classified as belonging to two phases, e.g., M/G1, meaning that the genes are maximally expressed in late M phase or at the M/G1 boundary. We plot the ‘cell cycle’ genes according to their POP coefficients in polar coordinates, i.e., (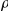, 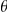), and mark different colors for different previous cell cycle classifications as shown in Figure 4. The median POP phases of the five experimentally defined clusters are 331.4° (S/G2), 266.6° (G2/M), 182.4° (M/G1), 102.0° (G1), and 32.4° (S). Genes that are experimentally classified as having maximal expression in the same phase of the cell cycle have similar POP phase values. Moreover, differences in POP phase correspond to different phases of the cell cycle. Thus, POP analysis provides an approach for clustering genes according to the peaks of the oscillation patterns of their expressions across the cell cycle.

### Genes consistently not identified as periodic across the cell cycle have low POP amplitudes

A key question is whether POP analysis is specific for identifying periodically expressed genes, i.e., does POP analysis falsely identify genes as periodic that are not actually periodically expressed? To address this question, we examine the results of POP analysis on genes that have never been identified by either previous experiments or existing computational methods as periodic across the cell cycle. Experimental methods for identifying genes periodically expressed across the cell cycle were introduced in [2], [5], [15], [16]. Existing computational methods for identifying ‘cell cycle’ genes for *α*-factor-based synchronization [1], [2], [3], [4], [5], [6] are summarized in [1]. Out of 4598 genes reported here, there are 3429 genes that have never been identified as periodically expressed across cell cycle by any of these previous studies.

The mean of the POP amplitudes of these 3429 ‘not cell cycle’ genes (0.064) is significantly (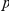
<0.01) smaller than the mean of the POP amplitudes of the remaining genes (0.096), according to a two-sample t-test with unequal variances. (Their variances are not statistically equivalent, F test, 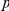
<0.01.)

### POP analysis identifies genes annotated in the ‘cell cycle’ category that are not identified as periodically expressed by experiments

In the preceding sections, we argued that POP analysis could identify ‘cell cycle’ and ‘non cell cycle’ genes consistent with previous experimental designations. Here we investigate whether POP analysis can provide insights that extend beyond what has already been reported from experimental investigations of gene expression across the cell cycle. Specifically, we ask, can POP analysis identify genes that are likely to be periodically expressed but that were missed in previously reported experiments? To address this question, we examine annotations [12] of genes that POP analysis identifies as periodically expressed across the cell cycle but that experimental methods do not identify as such.

**Figure 5:**
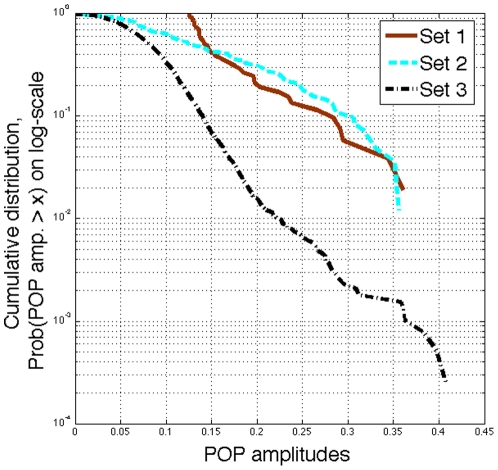
Cumulative distributions of POP amplitudes for demonstrating that previous experimental methods may miss ‘cell cycle’ genes. Cumulative distributions (Probability(POP amplitude >x)) in log-scale of POP amplitudes of three sets: Set 1 - 52 genes annotated as ‘cell cycle’ genes in MIPS but not identified as periodically expressed in reports of experimental methods [2], [5], [15], [16]; Set 2 - ‘cell cycle’ genes identified by experiments and also annotated in MIPS; Set 3 - genes that are not identified by experiments nor annotated in MIPS as ‘cell cycle’ genes.

As summarized in [1] the Munich Information Center for Protein Sequences (MIPS) [12] has annotated genes as belonging to various functional categories. The functional category ‘cell cycle’ includes genes involved in the transcription or regulation activity of cell cycle. Such genes may drive the oscillation processes in (14) so may have coherent, periodic expression across the cell cycle. We investigate the POP amplitudes of 52 genes annotated as ‘cell cycle’ genes in MIPS but not identified as periodically expressed in reports of experiments [2], [5], [15], [16] (Set 1 in Figure 5). The mean of the POP amplitudes of these 52 genes (0.14) is not statistically different from the mean of the POP amplitudes of ‘cell cycle’ genes identified by experiments and also annotated in MIPS (0.12) (Set 2 in Figure 5) based on a two-sample t-test with unequal variances (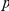 = 0.16). (Their variances are not statistically equivalent, F test, 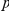
<0.01.) However, it must be acknowledged that the small sample size limits our ability to detect a statistically significant difference (post hoc power  = 0.29 for alpha  = 0.05). More important is the fact that the mean of the POP amplitudes of these 52 genes (0.14) is high, indicating that POP analysis concurs with their annotation as ‘cell cycle’ genes. Also, the mean POP amplitude of this group is significantly (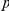
<0.01) larger than the mean of the POP amplitudes of genes that are not identified by experiments nor annotated in MIPS as ‘cell cycle’ genes (Set 3 in Figure 5) based on a two-sample t-test with unequal variances. (Their variances are not statistically equivalent, F test, 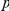
<0.01.) We plot the cumulative distributions [Probability(POP amplitude >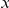)] on log-scale of POP amplitudes of these three sets as Figure 5. Thus, POP analysis is able to identify genes that may be ‘cell cycle’ genes, but which were not found in experiments.

### POP analysis identifies genes as unlikely to be periodically expressed that are not annotated as ‘cell cycle’ but are labeled as periodically expressed by experiments

A central question for POP analysis is whether it can be used to identify genes that may have been incorrectly labeled as being periodically expressed over the cell cycle based on reports of experimental methods. To address this question, we examine annotations [12] of genes that POP analysis identifies as probably not periodically expressed but that experimental methods identify as periodically expressed across the cell cycle.

There are 21 out of 344 experimentally identified ‘cell cycle’ genes that have particularly low POP amplitudes (lowest 10% of the set). We examine their MIPS annotations to evaluate the merits of the POP analysis for these genes. We find that MIPS does not annotate 16 out of these 21 genes (∼76%) as ‘cell cycle’ genes, which is consistent with the results of the POP analysis. These results indicate that experiments may mistakenly identify some genes as ‘cell cycle’ genes that can be correctly recognized as aperiodic with the assistance of POP analysis.

### Identification of cell cycle genes by POP amplitudes

A high POP amplitude indicates that a gene's expression is strongly periodic across the cell cycle. Thus far in our analyses, we have avoided applying arbitrary thresholds and have evaluated the POP approach based on comparison of distributions. However, in practical application of POP analysis to gene expression data, it would be valuable to apply a fixed threshold to denote some genes as ‘periodic’ and others as ‘aperiodic’. While such a threshold is necessarily data-set specific, we present a general approach for selecting an appropriate cutoff.

**Figure 6:**
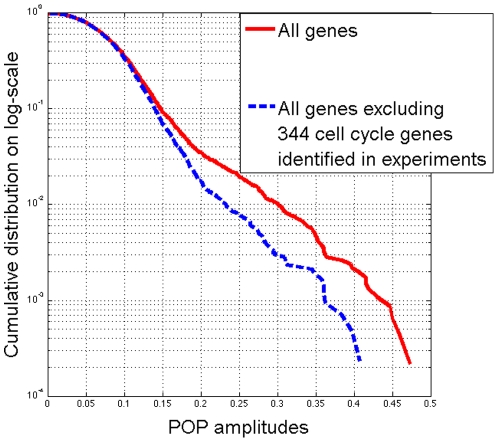
Cumulative distributions of POP amplitudes for selecting the POP amplitude threshold. Cumulative distributions (Probability(POP amplitude >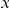)) on log-scale of POP amplitudes of all 4598 genes (solid curve) and all genes excluding 344 cell cycle genes identified in experiments (dashed curve). By visual inspection, we see that the gap between two curves starts at POP amplitude ≈ 0.1, which we consider is the cutoff. There are 846 genes whose POP amplitudes are greater than 0.1.

In order to choose a threshold on POP amplitudes to identify ‘cell cycle’ genes for the dataset with *α*-factor-based synchronization, we plot the cumulative distributions [Probability(POP amplitude >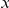)] on log-scale of POP amplitudes of all 4598 genes (solid curve) and all genes excluding 344 experimentally identified ‘cell cycle’ genes (dashed curve) as shown in Figure 6. By visual inspection, we see that the gap between the two curves starts at POP amplitude ≈ 0.1, which we take as the threshold for subsequent analysis. There are 846 genes in this data set whose POP amplitudes are greater than 0.1. A similar approach can be applied for other gene expression data sets in order to identify a data-set specific threshold for identifying genes with periodic expression.

### POP analysis compares favorably with existing computational methods for identifying ‘cell cycle’ genes and can potentially complement other approaches

In this assessment, we compare the sets of genes identified as periodically expressed across the cell cycle by POP analysis and six existing computational methods (Table S4). A detailed summary on these methods can be found in [1].

The overlap with the ‘cell cycle’ genes reported from experimental methods (all 465 genes) is also considered. We first compare POP with existing computational methods by analyzing the overlaps of the sets of genes identified by the different methods, and likewise the overlaps with the set of genes identified in reports of experiments. Then, we analyze the potential benefits of combining POP with existing methods.

### Comparative analysis

Each of the computational methods identifies a substantial number of genes as periodic that are not flagged by POP nor were reported as such from experiments (e.g., 371 for Spellman's method [5]). Likewise, POP analysis marks many genes as periodic that are not identified as such by the other computational methods considered nor by the experimental approach (e.g., 477 for POP analysis relative to Spellman's method). The same is true for the experimental approach; several genes are identified as periodic across the cell cycle that are not flagged by POP analysis or the other computational method considered (e.g., 221 for experiments relative to Spellman's method). However, there are also sizeable subsets wherein there is some agreement between the computational and experimental approaches. For example, Spellman's method and POP analysis both identify 137 genes as periodic across the cell cycle that are recorded as such based on experiments. Thus, there is a notably fraction of genes for which Spellman's method, POP analysis, and experiments agree. However, more interesting are the small subsets for which there is some but not universal agreement. For example, there are 208 genes flagged by both Spellman's and POP analysis that are not identified by experiments. Thus, we next investigate the potential benefits to be had from adding POP analysis to another computation method, such as Spellman's method.

### Complementary analysis

We analyze the potential benefits of combining the results of POP analysis and existing methods. We analyze Spellman's method as an example here, and provide similar comparison results for other computational methods in Figure S4 and Table S5.

Out of 800 genes identified by Spellman's method as periodically expressed over the cell cycle, 220 (28%) are also noted as such in reports of experiments. If we consider the 454 genes flagged by Spellman's method which are not flagged by POP analysis, the percentage that are also experimentally identified as ‘cell cycle’ drops to 18%. On the other hand, if we consider the 345 genes for which both Spellman and POP analysis indicate that they are likely to be periodically expressed, the percentage that are also experimentally identified as ‘cell cycle’ increases to 40%. Similar results are obtained when other computational methods are considered (data included in Table S4, Figure S4 and Table S5). These analyses suggest that combining POP analysis with other computational methods for identifying periodically expressed genes may increase the yield over those methods operating independently, as benchmarked against experimental methods.

## Discussion

In this paper, we have presented an application of POP analysis to genome-wide gene expression data. We model the genomic system using the first-order matrix ordinary-differential equation, which is known as the state equation in systems theory. Due to the small number of time samples and the huge number of genes in a typical genome-wide gene-expression data set, we first estimate the eigen-genomic system matrix that is much lower dimensional and more amenable to solve. The POPs of the eigen-genomic system are then identified. By multiplying the POPs of the eigen-genomic system by the coefficient matrix that maps between genes and eigengenes, we obtain the POPs of the genomic system.

We first evaluate the POPs using simulation data. The amplitudes and phases of the POPs extracted from the simulated data are found to highly correlate with the amplitudes and phases defined by the parameters of the simulation. Thus, POP analysis well recovers the amplitudes and phases of the oscillation processes that drive the periodical expressions of genes in simulation.

Real-world data, the budding yeast gene expression data with *α*-factor-based synchronization, is also used to evaluate POP analysis for gene expression data. The POP amplitudes of the cell cycle genes identified by previous experiments are found to be significantly greater than POP amplitudes of the rest of the genes. In addition, we demonstrate that the POP phases matched experimental cell cycle phase classifications. On average, the POP amplitudes of genes that have never been identified by either experiments or existing computational methods are significantly smaller than POP amplitudes of the rest of the genes. We find that POP analysis is able to identify possible ‘cell cycle’ genes that are not identified by experiments but that are annotated as ‘cell cycle’ genes. Moreover, we show that some genes identified in experiments as periodically expressed over the cell cycle had low POP amplitudes and are not annotated as ‘cell cycle’ genes. We also present a method to decide the threshold on the POP amplitude to identify genes as periodically expressed across the cell cycle. Previous experiments were implemented for individual genes rather than genome-wide, so they may not be accurate to reveal the dynamic characteristics of genes that are driven by genes' interactions at genome system-level, which might explain the discrepancies that POP analysis reports.

Finally, we demonstrate that combining the results of POP analysis with that of existing computational approaches for identifying periodically expressed genes has the potential to provide an increased yield relative to only using existing computational approaches. It is not unexpected that POP analysis provides complementary information relative to existing computational methods given that POP is a very different approach to identifying periodically expressed genes. A detailed summary on these methods can be found in [1]; we review only briefly here to explain how they are different from POP analysis. All six of the existing computational methods considered in this study analyze expression levels for individual genes only, as opposed to a system of genes, and identify periodic expression by fitting the individual gene expression level over time to common mathematical functions: sine functions [2], [3], [5], single-pulse models [6], and cubic splines [4]. However, the dynamic characteristics of a gene's expression are driven by interactions with other genes at genomic system-level; analyzing genes one-by-one cannot capture these interdependencies. The use of common functions enables straightforward mathematical analysis; however, they may not accurately capture the dynamic characteristics of a particular biological system. Moreover, fitting all genes to the same function may not unveil the dynamic characteristics of individual genes since the quality of the fit may vary from gene to gene. POP analysis, on the other hand, is a data-driven method that obtains the dynamic characteristics of each gene based on a genomic system-level model using systems theory.

We should point out that computationally classifying a gene as ‘cell cycle’ or not ultimately depends on the criteria used for quantifying the periodic expression, e.g., POP amplitude is the criterion in the proposed method. The value of an approach for classifying genes is ultimately determined by whether the method enables new insight into the biological system. Towards the goal, the choice of the threshold on the POP amplitude to define ‘cell cycle’ genes makes use of previously reported experimental results. Moreover, the comparison of the results of POP analysis, experiments, and a gene annotation database suggests that POP analysis may in fact enable scientific discovery, not merely reproduction of established knowledge.

The real-world dataset that we have used [5] is 13 years old and has been analyzed in hundreds of follow-up studies. We chose this well-studied data set precisely for this reason. The main point of our paper is to present a novel method, one that we believe can identify cell-cycle genes in a better and more systematic way than previous methods. By using a well-understood data set as the basis for our analysis, we have a solid reference of previous works against which we can compare our results. However, in order to verify our findings, we have also applied POP analysis to a newer dataset, Alpha 30 in [18]. This dataset includes 4774 genes at time, 0, 5, 10, … , 120 minutes. Out of those 4774 genes in the Alpha 30 data set, there are 3512 genes (74%) reported in the Spellman's dataset that we mainly applied above. We obtained similar results with the Alpha 30 data set. The first and second complex conjugate eigenvalues correspond to POPs with a period of 69 minutes, which falls into the estimated period of the cell cycle (66±11 minutes) of budding yeast with *α*-factor-based synchronization [5], [14]. We include POP amplitudes and phases in Table S6. The mean of the POP amplitudes of experimentally identified ‘cell cycle’ genes is significantly (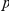
<0.01) greater than the mean of the POP amplitudes of the rest of the genes according to both a two-sample t-test with unequal variances and a permutation test. The POP amplitude threshold ≈ 0.04, and there are 478 genes whose POP amplitudes are greater than this threshold. Out of those 478 genes, there are 202 genes (42%) also identified by our method using the Spellman's dataset.

There are limitations to the application of POP analysis to gene expression data. In particular, POP analysis requires that the time samples be taken as equal intervals. Thus, POP analysis cannot be applied to genome-wide gene expression data that were sampled at unequal time intervals, which is a common experimental design. One possible strategy to overcome this limitation would be to interpolate the data first and then sample at equally-timed intervals [9]. It should also be noted that whereas existing computational approaches for identifying periodically expressed genes consider each gene one-by-one, POP analysis of genome-wide expression is open to the opposite criticism; that is, when POP analysis is applied to whole-genome data, the expression level of each gene is modeled as possibly being dependent on the expression level of all other genes. In reality, the expression level of a given gene likely depends upon the expression levels of some subset of the genome. In future work, POP analysis could potentially be combined with data mining techniques to create more biologically plausible models of periodic gene expression that employ a flexible system size that may be smaller than the whole genome.

In conclusion, we present the first application of POP analysis, a multivariate and systematic method, to genome-wide gene expression data to identify genes that are periodically expressed across the cell cycle. Using both simulation and real-world data, we show that POP analysis is not only compares favorably with experiments and existing computational methods, but that it also provides complementary information relative to other approaches.

## Supporting Information

Figure S1
**Dynamic trajectory of principal oscillation patterns (POPs).** The POPs, 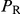 and 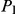, two *N*-dimensional vectors, drive the cosine and sine, respectively, of the oscillation part with angular frequency *ω*. The oscillation part of an *N*-dimensional genomic system starts from 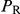 to 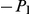 to 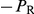 to 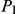, and then back to 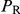 with period 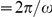.(DOC)

Figure S2
**Scatter plot and Pearson correlation of POP amplitudes vs. Simulated amplitudes.** The Pearson correlation between the gene expression amplitudes defined in the simulation and the amplitudes recovered by POP analysis is 0.99 with 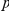
<0.01. Thus, the POP amplitudes well recover the simulation oscillation amplitudes.(DOC)

Figure S3
**Scatter plot and Pearson correlation of POP phases vs. Simulated phases.** There is high Pearson correlation between the gene expression phases defined in the simulation and the phases recovered by POP analysis (rho  = 0.96 with 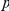
<0.01 for sine values). So, the POP phase of a simulated gene reflects the phase of the oscillation process in its expression.(DOC)

Figure S4 **‘Cell cycle’ genes identified by Experiment vs. POP vs. Existing methods.** Venn diagrams show overlaps of ‘cell cycle’ genes identified by previous experiments, POP analysis and each existing computational method.
(DOC)

Table S1 **Eigenvalues and POP period of simulated genomic system.**
(DOC)

Table S2 **Eigenvalues and POP period of the genomic system of the budding yeast with** ***α*** **factor-based synchronization.**
(DOC)

Table S3 **POP amplitudes and phases of all 4598 genes.**
(XLS)

Table S4 **‘Cell cycle’ genes identified by POP, experiment, MIPS and existing computational methods.**
(XLS)

Table S5 **Overlap in percentage of experimentally identified ‘cell cycle’ genes identified by POP and existing methods.**
(XLS)

Table S6 **POP amplitudes and phases of all 4774 genes in Dataset alpha 30,** http://labs.fhcrc.org/breeden/cellcycle/ **.**
(XLS)
