# Supplementary material for: Principal-Oscillation-Pattern Analysis of Gene Expression
Source: PLoS One. 2012 Jan 10;7(1):e28805. doi: 10.1371/journal.pone.0028805 (PMC3254616; doi:10.1371/journal.pone.0028805)
Supplement: Figure S1 — Dynamic trajectory of principal oscillation patterns (POPs). The POPs, and , two N-dimensional vectors, drive the cosine and sine, respectively, of the oscillation part with angular frequency ω. The oscillation part of an N-dimensional genomic system starts from to to to , and then back to with period . (DOC) [file pone.0028805.s001.doc]

| 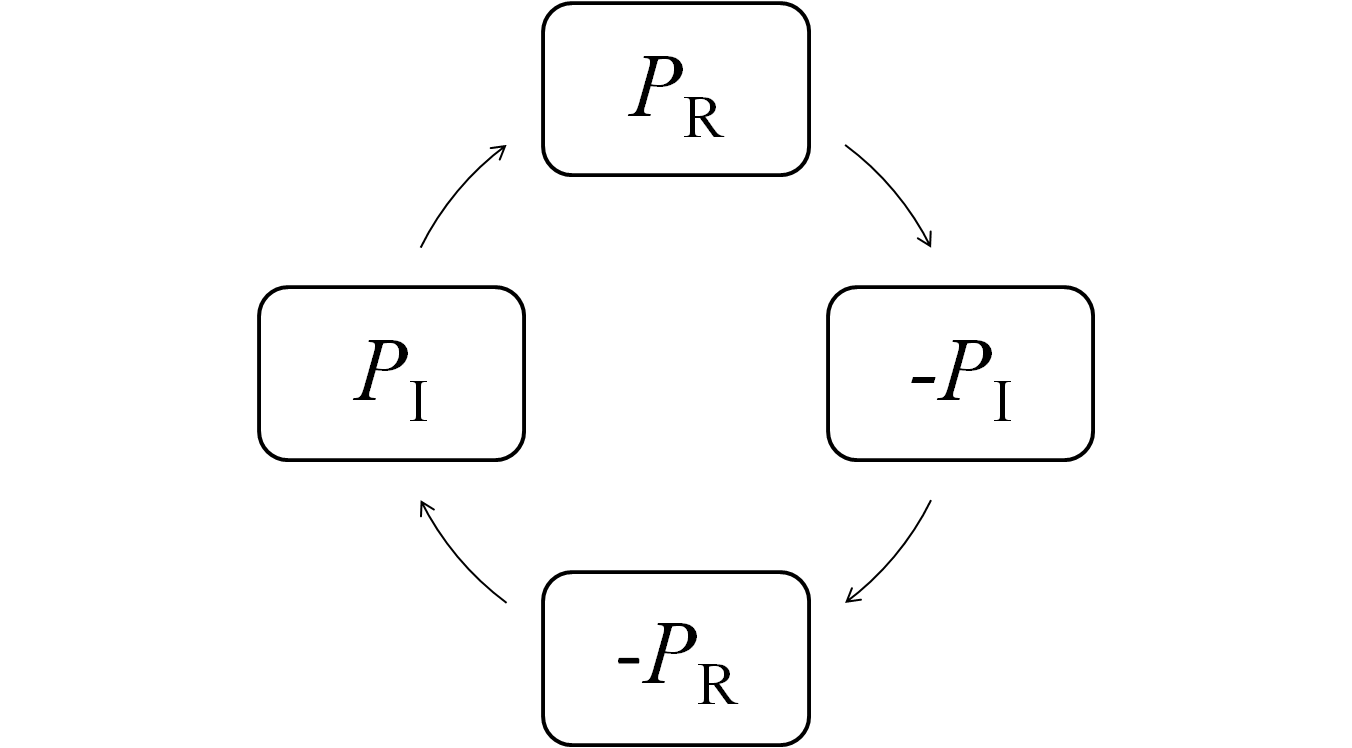 |
| --- |
| **Figure S1**. **Dynamic trajectory of principal oscillation patterns (POPs).** The POPs, *P*_R_ and *P*_I_, two *N*-dimensional vectors, drive the cosine and sine, respectively, of the oscillation part with angular frequency *ω*. The oscillation part of an *N*-dimensional genomic system starts from *P*_R_ to *−P*_I_ to *−P*_R_ to *P*_I_, and then back to *P*_R_ with period =*2π/ω*. |
