# Supplementary material for: Principal-Oscillation-Pattern Analysis of Gene Expression
Source: PLoS One. 2012 Jan 10;7(1):e28805. doi: 10.1371/journal.pone.0028805 (PMC3254616; doi:10.1371/journal.pone.0028805)
Supplement: Figure S2 — Scatter plot and Pearson correlation of POP amplitudes vs. Simulated amplitudes. The Pearson correlation between the gene expression amplitudes defined in the simulation and the amplitudes recovered by POP analysis is 0.99 with <0.01. Thus, the POP amplitudes well recover the simulation oscillation amplitudes. (DOC) [file pone.0028805.s002.doc]

| 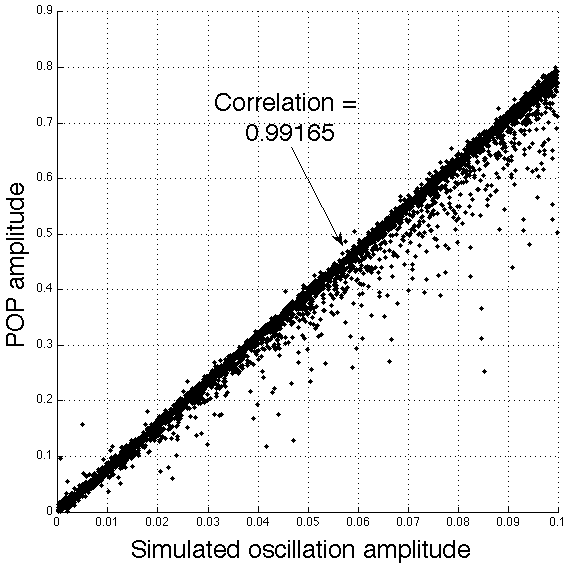   |  | | --- | | **Figure S2.** **Scatter plot and Pearson correlation of POP amplitudes vs. Simulated amplitudes.** The Pearson correlation between the gene expression amplitudes defined in the simulation and the amplitudes recovered by POP analysis is 0.99 with *p* < 0.01. Thus, the POP amplitudes well recover the simulation oscillation amplitudes. | |  | |
| --- | --- | --- | --- |
|  |
