# Supplementary material for: Principal-Oscillation-Pattern Analysis of Gene Expression
Source: PLoS One. 2012 Jan 10;7(1):e28805. doi: 10.1371/journal.pone.0028805 (PMC3254616; doi:10.1371/journal.pone.0028805)
Supplement: Figure S3 — Scatter plot and Pearson correlation of POP phases vs. Simulated phases. There is high Pearson correlation between the gene expression phases defined in the simulation and the phases recovered by POP analysis (rho = 0.96 with <0.01 for sine values). So, the POP phase of a simulated gene reflects the phase of the oscillation process in its expression. (DOC) [file pone.0028805.s003.doc]

| 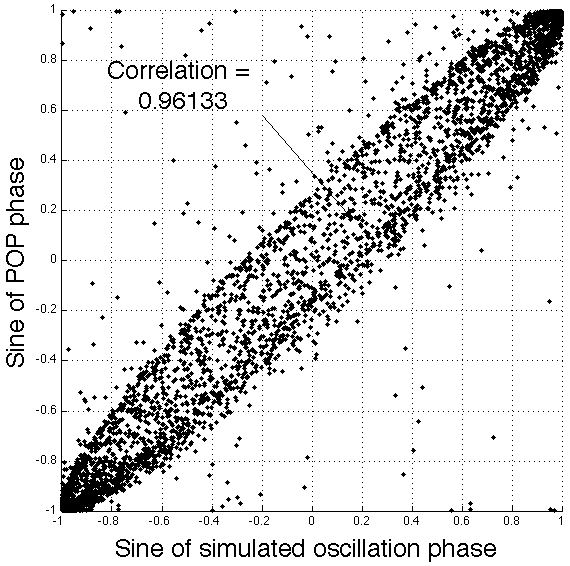 |
| --- |
| **Figure S3.**  **Scatter plot and Pearson correlation of POP phases vs. Simulated phases.** There is high Pearson correlation between the gene expression phases defined in the simulation and the phases recovered by POP analysis (rho = 0.96 with *p* < 0.01 for sine values). So, the POP phase of a simulated gene reflects the phase of the oscillation process in its expression. |
