# Supplementary material for: Principal-Oscillation-Pattern Analysis of Gene Expression
Source: PLoS One. 2012 Jan 10;7(1):e28805. doi: 10.1371/journal.pone.0028805 (PMC3254616; doi:10.1371/journal.pone.0028805)
Supplement: Figure S4 — ‘Cell cycle’ genes identified by Experiment vs. POP vs. Existing methods. Venn diagrams show overlaps of ‘cell cycle’ genes identified by previous experiments, POP analysis and each existing computational method. (DOC) [file pone.0028805.s004.doc]

| (a)  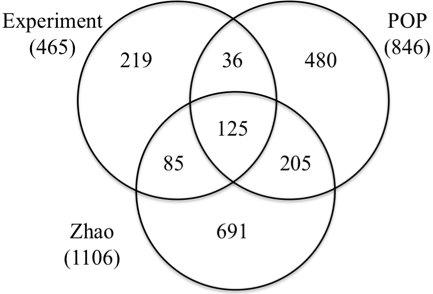 | (b)  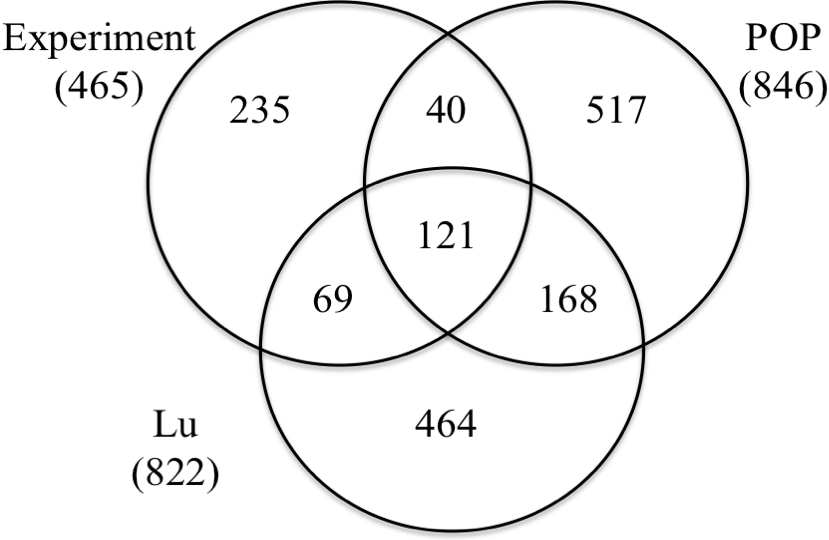 |
| --- | --- |
| (c)  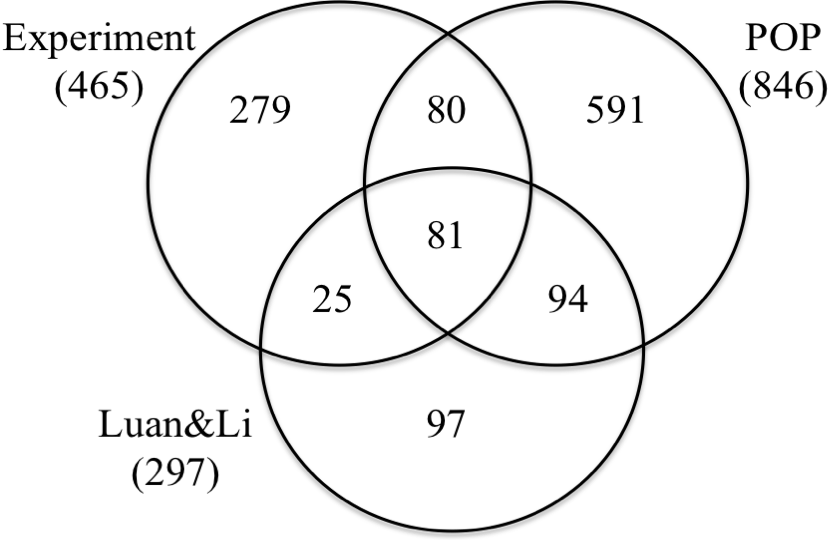 | (d)  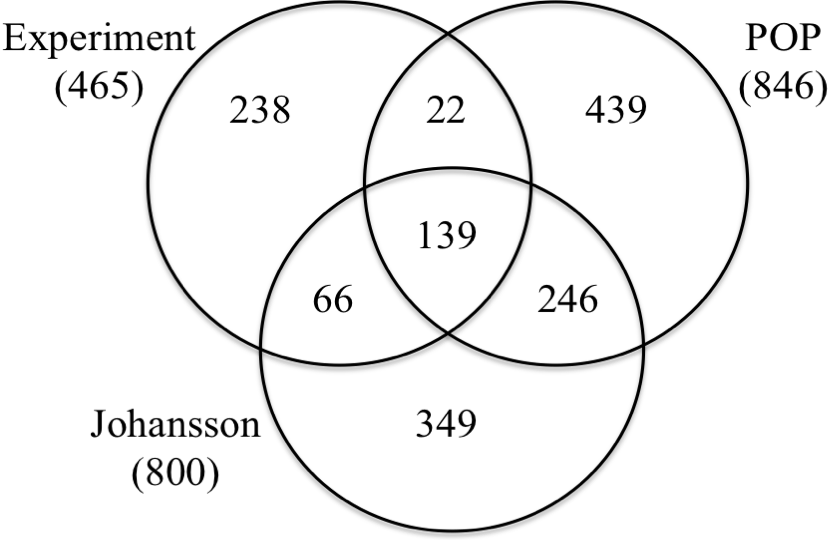 |
| (e)  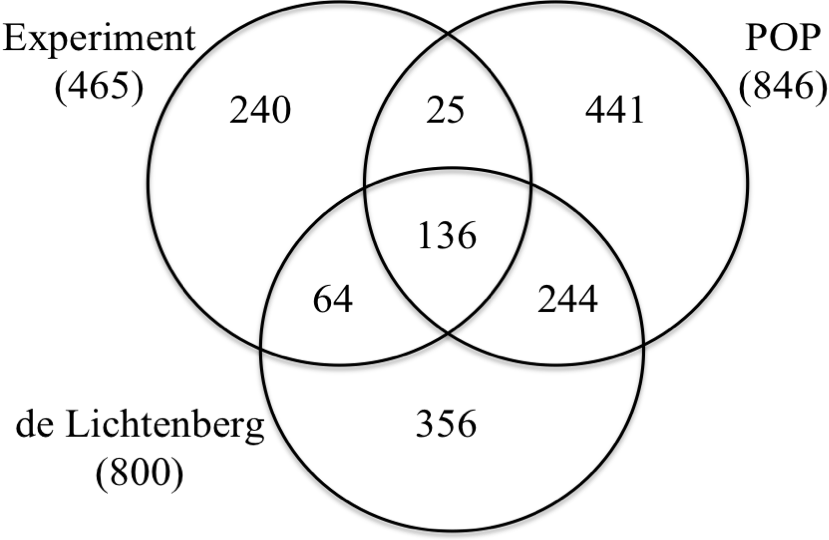 | (f)  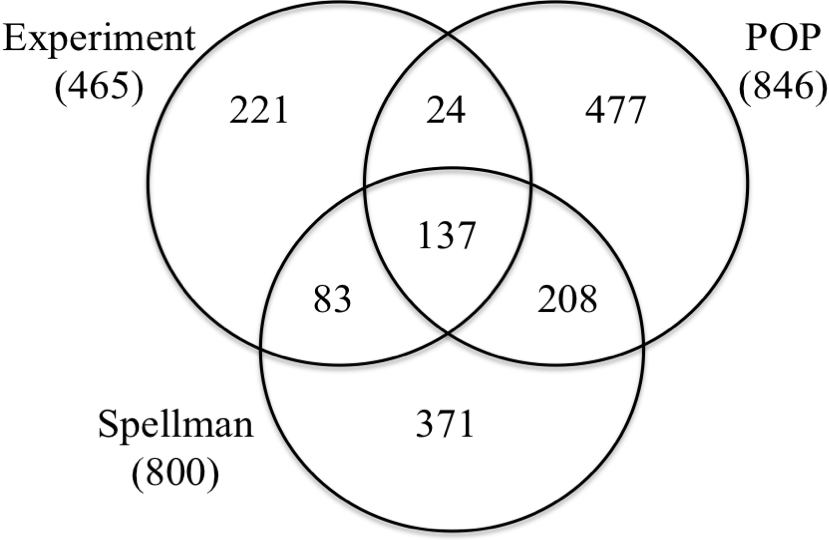 |

**Figure S4**. **‘Cell cycle’ genes identified by Experiment vs. POP vs. Existing methods.** Venn diagrams show overlaps of ‘cell cycle’ genes identified by traditional experiments, POP analysis and each existing computational method. (a) Zhao LP, Prentice R, Breeden L (2001) Statistical modeling of large microarray data sets to identify stimulus-response profiles. Proc Natl Acad Sci U S A 98: 5631-5636. (b) Lu X, Zhang W, Qin ZS, Kwast KE, Liu JS (2004) Statistical resynchronization and Bayesian detection of periodically expressed genes. Nucleic Acids Res 32: 447-455. (c) Luan Y, Li H (2004) Model-based methods for identifying periodically expressed genes based on time course microarray gene expression data. Bioinformatics 20: 332-339. (d) Johansson D, Lindgren P, Berglund A (2003) A multivariate approach applied to microarray data for identification of genes with cell cycle-coupled transcription. Bioinformatics 19: 467-473. (e) de Lichtenberg U, Jensen LJ, Fausboll A, Jensen TS, Bork P, et al. (2005) Comparison of computational methods for the identification of cell cycle-regulated genes. Bioinformatics 21: 1164-1171. (f) Spellman PT, Sherlock G, Zhang MQ, Iyer VR, Anders K, et al. (1998) Comprehensive identification of cell cycle-regulated genes of the yeast Saccharomyces cerevisiae by microarray hybridization. Mol Biol Cell 9: 3273-3297.
