# Supplementary material for: Principal-Oscillation-Pattern Analysis of Gene Expression
Source: PLoS One. 2012 Jan 10;7(1):e28805. doi: 10.1371/journal.pone.0028805 (PMC3254616; doi:10.1371/journal.pone.0028805)
Supplement: Table S1 — Eigenvalues and POP period of simulated genomic system. (DOC) [file pone.0028805.s005.doc]

**Table S1.** Eigenvalues and POP period of simulated genomic system.

| Eigenvalue # | 1/2 | 3 | 4 | 5 |
| --- | --- | --- | --- | --- |
| ln(Eigenvalue) | ±0.2093*j* | -0.0101 | -0.0335 | -0.1068 |
| Period (minute) | 30 | - | - | - |
