# Supplementary material for: Principal-Oscillation-Pattern Analysis of Gene Expression
Source: PLoS One. 2012 Jan 10;7(1):e28805. doi: 10.1371/journal.pone.0028805 (PMC3254616; doi:10.1371/journal.pone.0028805)
Supplement: Table S2 — Eigenvalues and POP period of the genomic system of the budding yeast with α factor-based synchronization. (DOC) [file pone.0028805.s006.doc]

**Table S2.** Eigenvalues and POP period of the genomic system of the budding yeast with *α* factor-based synchronization.

| Eigenvalue # | 1/2 | 3 | 4 | 5 |
| --- | --- | --- | --- | --- |
| ln(Eigenvalue) | −0.0610 ± 0.0956*j* | -0.0076 | -0.0909 | -0.2282 |
| Period (minute) | 65.7 | - | - | - |
